# Pneumococcal Serotype–specific Unresponsiveness in Vaccinated Child with Cochlear Implant

**DOI:** 10.3201/eid1806.110906

**Published:** 2012-06

**Authors:** Elaine Stanford, Shamez Ladhani, Mary Slack, David Scott, Alec Fitzgerald-O’Connor, Pauline Waight, Rashmi Malkani, Ray Borrow

**Affiliations:** Health Protection Agency, Manchester, UK (E. Stanford, R. Borrow);; Health Protection Agency, London, UK (S. Ladhani, M. Slack, P. Waight, R. Malkani);; East Sussex Hospitals National Health Service Trust, Conquest Hospital, St Leonards-on-Sea, UK (D. Scott);; St Thomas’ Hospital London, London (A. Fitzgerald-O’Connor);; University of Manchester, Manchester (R. Borrow)

**Keywords:** invasive pneumococcal disease, recurrent, cochlear implant, serotype hyporesponsiveness, pneumococcal conjugate vaccination, bacteria

**To the Editor**: Approximately 100,000 persons worldwide have received cochlear implants for hearing loss, and more children now receive them than ever (*1*). Such children have a >30-fold increased risk for pneumococcal meningitis than the background rate (*1**,**2*). During 2006–2010, children born in the United Kingdom were offered the 7-valent pneumococcal conjugate vaccine (PCV7) at 2, 4, and 13 months of age (*3*). Those at high risk for invasive pneumococcal disease (IPD) were additionally offered the 23-valent pneumococcal polysaccharide vaccine (PPV23) at 2–5 years (*3*). We describe a fully vaccinated child with a cochlear implant in whom recurrent pneumococcal meningitis developed, caused by a vaccine serotype (i.e., vaccine failure). The child continues to have nonprotective antibody concentrations against the infecting serotype, despite further pneumococcal vaccination.

A previously healthy, appropriately vaccinated 23-month-old girl (Table) had a cochlear device implanted in the right ear after receiving (through the universal newborn hearing screening program) a diagnosis of profound, bilateral, sensorineural deafness. Two weeks later, she exhibited fever, lethargy, and drowsiness. On hospital admission, she had a peripheral blood leukocyte count of 19.3 × 10^9^ cells/L, a neutrophil count of 17.0 × 10^9^ cells/L, and C-reactive protein level 75 mg/L. Meningitis was diagnosed, and she received intravenous ceftriaxone but was too ill for a lumbar puncture. Blood cultures subsequently grew fully sensitive *Streptococcus pneumoniae*, later confirmed as serotype 4 by the national reference laboratory. She was discharged after 14 days of receiving intravenous antimicrobial drugs without complications.

**Table:** . Pneumococcal serotype-specific IgG concentrations in 2-year-old child with recurrent pneumococcal meningitis, United Kingdom*

Patient age, mo	Event	Serotype-specific IgG, µg/mL						
		4	6B	9V	14	18C	19F	23F
1.9	PCV7 dose 1							
3.9	PCV7 dose 2							
13.4	PCV7 dose 3							
22.8	Cochlear implant							
23.4	Pneumococcal meningitis (episode 1)							
24.4	PCV7 dose 4							
25.6	Pneumococcal serologic testing	0.12	27.6	23.0	36.9	13.8	57.0	41.1
27.8	PPV23 dose 1							
28.8	Pneumococcal serologic testing	0.18	14.2	13.1	30.4	11.4	11.7	32.0
32.0	Pneumococcal meningitis (episode 2)							
35.1	PCV7 dose 5							
36.1	Pneumococcal serologic testing	0.20	18.40	7.12	12.50	12.40	2.78	37.1
39.0	PCR positive nasopharyngeal swab specimen for *Streptococcus pneumoniae* serotype 4							

*PCV7, 7-valent pneumococcal conjugate vaccine; PPV23, 23-valent pneumococcal polysaccharide vaccine. Blank spaces indicate not tested.

At 24 months, she received a fourth dose of PCV7. Blood tests 1 month later showed good antibody responses to 6 PCV7 serotypes but not to serotype 4, which did not reach the putative protective level of >0.35 µg/mL antibody threshold (Table). At 28 months, she received 1 dose of PPV23 per national guidelines (*3*). Four months later, she was brought to the hospital with fever, rigors, drowsiness, and vomiting. Blood tests showed a leukocyte count of 24.4 × 10^9^ cells/L, neutrophil count of 21.6 × 10^9^ cells/L, and C-reactive protein level of 272 mg/L. Lumbar puncture performed the next day showed 890 leukocytes/mL (predominantly polymorphs), cerebrospinal fluid glucose level <1.1 mmol/L, protein level of 1.0 g/L, gram-positive diplococci on Gram staining, and positive PCR results for pneumococci, although cerebrospinal fluid culture was negative.

A blood culture grew fully sensitive *S. pneumoniae*, also confirmed by the national reference laboratory as serotype 4. She recovered after receiving intravenous ceftriaxone and oral rifampin for 2 weeks, followed by 4 weeks of oral amoxicillin and rifampin. She then received prophylactic oral penicillin for maintenance. Subsequently, an abdominal ultrasound confirmed the presence of a spleen, and her immunoglobulin concentrations were in the normal range. At 35 months, she received another dose of PCV7, and a blood test 1 month later showed variable but high responses to 6 of the PCV7 serotypes and no response to serotype 4 (Table). Moreover, nasopharyngeal swab specimens, obtained when the patient was 39 months old and receiving penicillin prophylaxis, were positive for serotype 4.

We described 8 previously healthy children with serotype-specific immune unresponsiveness after IPD, although a second IPD episode did not develop in these children (*4*). This phenomenon may result from large pneumococcal polysaccharide loads that deplete the memory B-cell pool and cause immune paralysis (*4**,**5*). In immunogenicity studies, some infants (1%–3%) remain unresponsive to conjugate vaccines (*5*). In a randomized controlled trial of PPV23 in 50–85-year-old persons, 3 vaccinated persons with culture-confirmed IPD had adequate pre- and postvaccination antibody concentrations to all but the infecting serotype, suggesting that they were unresponsive to the infecting serotype before vaccination (*6*). In infants, recent randomized controlled trials have found that nasopharyngeal carriage at first dose of PCV7 resulted in significantly lower IgG responses to that specific serotype than occurred with noncarriers or carriers of other serotypes, possibly because of high carriage–induced polysaccharide loads (*7**,**8*). Moreover, unresponsiveness was only partially overcome by the 12-month PCV7 booster (*7*).

This case raises key questions regarding long-term clinical management of children with serotype-specific immune unresponsiveness after vaccination or infection. The case is further complicated by the patient’s cochlear implant, which may have been the source of infection (*9*), as well as evidence of nasopharyngeal carriage while the patient was receiving antimicrobial drug prophylaxis and recurrence of meningitis caused by the same serotype. However, her ability to respond to the other 6 PCV7 serotypes, normal immunoglobulin concentrations, no previous history of recurrent infections, and presence of a spleen all provide evidence against an underlying immune problem.

Further pneumococcal vaccination of this patient is unlikely to reverse the unresponsiveness, which may persist for years (*4**,**5*). Studies to clarify the immune mechanisms underlying unresponsiveness and strategies to reverse this phenomenon are, therefore, urgently warranted. In the meantime, we recommend that the infecting pneumococcal serotype be determined in children with IPD and that, when possible, those infected with a vaccine-related strain (particularly children with risk factors) have serotype-specific pneumococcal antibodies measured after infection. Appropriate measures to prevent recurrent IPD should also be taken, such as removal of potentially infected devices or long-term prophylaxis with antimicrobial drugs.
